# Motives for Participating in a Web-Based Nutrition Cohort According to Sociodemographic, Lifestyle, and Health Characteristics: The NutriNet-Santé Cohort Study

**DOI:** 10.2196/jmir.3161

**Published:** 2014-08-07

**Authors:** Caroline Méjean, Fabien Szabo de Edelenyi, Mathilde Touvier, Emmanuelle Kesse-Guyot, Chantal Julia, Valentina A Andreeva, Serge Hercberg

**Affiliations:** ^1^Université Paris 13, Sorbonne Paris Cité, Equipe de Recherche en Epidémiologie Nutritionnelle (EREN), Centre d’Epidémiologie et Biostatistiques Paris Nord, Inserm (U1153), Inra (U1125), Cnam, Université Paris 5, Université Paris 7BobignyFrance

**Keywords:** Internet, cohort study, population characteristics, motivation, participation

## Abstract

**Background:**

In traditional epidemiological studies, participants are likely motivated by perceived benefits, feelings of accomplishment, and belonging. No study has explored motives for participation in a Web-based cohort and the associated participant characteristics, although such information is useful for enhancing recruitment and improving cohort retention.

**Objective:**

We aimed to evaluate the relationships between motives for participation and sociodemographic, health, and lifestyle characteristics of participants in the NutriNet-Santé Web-based cohort, designed to identify nutritional risk or protective factors for chronic diseases.

**Methods:**

The motives for participation were assessed using a specifically developed questionnaire administered approximately 2 years after baseline. A total of 6352 completed the motives questionnaire (43.34%, 6352/15,000 randomly invited cohort participants). We studied the associations between motives (dependent variables) and individual characteristics with multivariate multinomial logistic regression models providing odds ratios and 95% confidence intervals.

**Results:**

In total, 46.45% (2951/6352) of participants reported that they would not have enrolled if the study had not been conducted on the Internet, whereas 28.75% (1826/6352) were not sure. Men (OR 1.21, 95% CI 1.04-1.42), individuals aged 26-35 years (OR 1.51, 95% CI 1.20-1.91), and obese participants (OR 1.30, 95% CI 1.02-1.65) were more inclined to be motivated by the Internet aspect. Compared with younger adults and managerial staff, individuals >55 years (OR 0.60, 95% CI 0.48-0.45) and employees/manual workers were less likely motivated by the Internet aspect (OR 0.77, 95% CI 0.63-0.92). Regarding reasons for participation, 61.37% (3898/6352) reported participating to help advance public health research on chronic disease prevention; 22.24% (1413/6352) to help advance nutrition-focused research; 6.89% (438/6352) in response to the call from the media, after being encouraged by a close friend/associate, or a medical provider. Individuals >45 years (vs younger participants) were more likely (OR 1.62, 95% CI 1.07-2.47), whereas overweight and obese participants (vs nonobese participants) were less likely to participate in the study for reasons related to helping public health research on chronic disease prevention (OR 0.72, 95% CI 0.58-0.89; OR 0.62, 95% CI 0.46-0.84; respectively). Exclusive public funding of the study was important for 67.02% (4257/6352) of the participants. Men (OR 1.37, 95% CI 1.17-1.61) and persons >55 years (OR 1.97, 95% CI 1.57-2.47) were more likely to consider the exclusive public funding as very important.

**Conclusions:**

The use of the Internet, the willingness to help advance public health research, and the study being publicly funded were key motives for participating in the Web-based NutriNet-Santé cohort. These motives differed by sociodemographic profile and obesity, yet were not associated with lifestyle or health status. These findings can help improve the retention strategies in Web-based cohorts, particularly during decisive study periods when principal exposure information is collected.

## Introduction

The successful implementation of very large population-based cohort studies involving collection of comprehensive, high-quality dietary, lifestyle, and health data is both a priority and a challenge in nutritional epidemiology [1,2]. Such observational studies face very high logistic costs and require substantial resources. The rapid and widespread increase in access to Internet has made this tool a viable and logical base for cohort studies because it presents advantages across all research stages [3,4]. In most industrialized countries, Internet access is greater than 50% and is still increasing [5]. In Europe, Internet users are becoming more representative of the general population, including older adults (42% of individuals older than 55 years are regular users) and people of low socioeconomic status (73% of individuals belonging to low socioprofessional categories are regular users) [6]. In France, in November 2009, 34.7 million French citizens (approximately 65 % of the population older than 11 years) were connected to the Internet during the previous month [7].

Yet Web-based prospective cohort studies are still in their infancy [8]. Whereas issues related to participation are crucial in epidemiological studies [9], participation in both Web-based cohorts and repeated-measures cross-sectional studies, and associated sociodemographic profiles have been rarely investigated [8,10-22]. A few studies have compared the sociodemographic and lifestyle characteristics of participants who used Web-based questionnaires with paper-and-pencil respondents [8,14]. The Black Women’s Health Study showed that Web-based questionnaire users were younger and had higher socioeconomic status than paper-and-pencil users, but no difference was observed in terms of lifestyle or health status [14]. These authors underlined the fact that low socioeconomic status and older age, which are markers of Web access, remain barriers to the use of Web-based questionnaires. In turn, the Millennium cohort study with military personnel has highlighted that Web responders were more likely to be male, younger, highly educated, obese, and smokers than were paper-and-pencil responders [8]. The results regarding sex, age, and educational level seem to be due to greater technological competence in these groups and greater access to the Internet, whereas findings on weight status and smoking may reflect subtle occupational differences, such as being employed in a sedentary work environment or an unhealthier lifestyle outside of work. Also, the ATTEMPT cohort has shown that participants had similar sociodemographic and lifestyle profiles to those found in the general population [16], whereas NINFEA cohort participation, regarding Italian pregnant women, was associated with being older, having a higher educational level, and being native Italian compared to the general population, probably due to population-specific occurrence [13]. In Web-based intervention studies with repeated measurements, women, older participants, nonsmokers, heavy alcohol consumers, and overweight participants were more likely to remain enrolled in Internet-delivered behavior change programs [17-22].

Voluntariness refers to the voluntary motivational nature of a person’s participation from the initial decision to participate through the course of the study, and is influenced by external and internal factors [23]. In traditional epidemiological studies, participants are motivated by the benefits they perceive, particularly the information and care received during the medical examinations, the positive feelings about oneself or an enhanced self-image generated by the act of participation, and the sense of loyalty and belonging associated with being part of the study [24-28]. To our knowledge, no study has explored motives for participation in a Web-based cohort and the associated participant characteristics, particularly during key periods of collection of exposure data. Such information would be useful in enhancing the recruitment of diverse population samples and in improving cohort retention.

The NutriNet-Santé study was launched in May 2009 in France to investigate multiple facets of the relationship between nutrition and health along with determinants of dietary behavior [29]. This is a large, Web-based, prospective, nutritional epidemiology cohort. To date, 156,734 volunteers aged >18 years have been included in the cohort. Participants are followed via a website specifically created for that purpose. The objective of the present study was to assess motives for participation in the NutriNet-Santé cohort after 2 years of participation, such as reasons for participation, the influence of the Internet support in participation, and the importance of public funding. We also explored sociodemographic, lifestyle, and heath characteristics associated with those motives.

## Methods

### Population

Participants were part of the NutriNet-Santé Study, a large Web-based prospective observational cohort. It is implemented in a general population targeting Internet-using adult volunteers aged 18 years and older. The design, methods, and rationale have been described elsewhere [29]. Eligible participants were recruited by different means. Initially, a vast multimedia campaign (television, radio, national and regional newspapers, posters, and Internet) called for volunteers and provided details about the study’s website [12]. The multimedia campaigns are repeated every 6 months. Recruitment information was maintained on a large number of websites (national institutions, city councils, private firms, Web organizations) and is regularly updated via professional channels (eg, doctors, pharmacists, dentists, business partners, municipalities). The key message delivered in the call for volunteers was:

Numerous scientific studies have highlighted the role of nutrition as a protective factor or a risk of many common diseases in France, as in all industrialized countries, such as cancer, cardiovascular diseases, obesity, type 2 diabetes, dyslipidemia, and hypertension. Nutrition is not the only determinant of these health problems. Indeed, genetic, biological, and environmental factors are involved in the onset of these diseases. To highlight the specific role of nutritional factors in health, the development of cohort studies with very large populations (group of participants followed for several years) is essential as they permit to accurately measure food intake, but also take into account other determinants, such as physical activity, weight, smoking, and family history of disease. The purpose of our study is to identify nutritional risk factors or protective factors for these diseases, which is an essential step in establishing dietary recommendations to prevent the risk of disease and improve the health of the current and future generations. This is the ambitious goal of the NutriNet-Santé study and that is why researchers need you.

During each multimedia recruitment campaign and during the enrollment process, participants are informed that follow-up over at least 10 years is planned.

Previous findings showed that most of the participants enrolled after hearing about the study on television because this medium entails the widest reach [12]. In particular, television announcements permitted the recruitment of members of population subgroups that are not typically well represented in population-based epidemiological research, those belonging to lower socioeconomic strata. The radio, newspapers, Internet, and advice from acquaintances also proved to be substantial means of disseminating information about this epidemiological study to encourage participation.

To be included, participants have to fill in on the website an initial set of questionnaires assessing dietary intake, physical activity, anthropometrics, lifestyle, and socioeconomic conditions along with health status. Participants were informed by email that, after inclusion, they would be asked to complete the same questionnaires each year as part of their follow-up. In addition, they are invited to fill in a complementary questionnaire each month. Aspects related to convenience of participation (ie, ≤20 min each month) and confidentiality were also emphasized. In addition, a system of boosting motivation and retention was implemented. In order to forge a sense of community that helps advance research, participants receive a NutriNet-Santé membership card at inclusion and a certificate on completion of each follow-up year/wave. They also receive monthly email with scientific information regarding health and nutrition, and invitations to press conferences about the study results. For purposes of retention, free screening tests for cholesterol, triglycerides, and diabetes are offered to participants (the results are sent back with a special notice in case of abnormal test results).

All baseline questionnaires were first pilot-tested and compared with traditional administration methods (paper-and-pencil versions or interviews by a dietitian) [30-32]. Health events are monitored through questionnaires about hospitalizations and medication use as well as via a linkage with the national vital statistics database. In April 2011, 15,000 randomly selected participants among 86,652 individuals included at the time of the study were invited to complete a Web-based questionnaire regarding their motives for participation in the study.

This study was conducted according to guidelines laid down in the Declaration of Helsinki, and all procedures were approved by the Institutional Review Board of the French Institute for Health and Medical Research (IRB Inserm no: 0000388FWA00005831) and the Commission Nationale Informatique et Libertés (CNIL no: 908450 and no: 909216). Written electronic informed consent to participate in the study was obtained from all participants.

### Data Collection

#### Motives for Participation

Participants were asked, “Would you have participated in the NutriNet-Santé study if it were not Internet-based?” (response options: yes, no, I don’t know). We also asked the participants, “What was your main reason for participating in the NutriNet-Santé study?” The response options for the different motives were classified into 2 general categories: (1) intrinsic motives for participation, including, to help advance public health research on chronic disease prevention, to help advance nutrition research, to receive regular scientific information about health and nutrition, out of curiosity, to belong to a group, or other motives and (2) extrinsic motives, including in response to the call from the media, from a close friend/associate, or from a medical provider. Finally, we asked participants, “Is the fact that the study is exclusively funded by public sources important for your participation?” (response options: very important, important, not very important, not important).

#### Assessment of Individual Characteristics

At baseline, sociodemographic, lifestyle, and health characteristics were self-reported. Participants indicated their alcohol consumption frequency and quantity over the previous 7 days. Alcohol intake was calculated by multiplying the alcohol content (ie, percentage) of each beverage (wine, beer, spirits, and cider) by the standard ethanol weight content. Body mass index (BMI) was assessed using self-reported height and weight. Status regarding type 2 diabetes, hypertension, and hypercholesterolemia was provided by participants by answering the following question: “Have you been or are you currently being treated for type 2 diabetes / hypertension / hypercholesterolemia?” If the participant answered yes, he/she completed the information by self-reporting the year of diagnosis and current use of medication.

### Statistical Analysis

The present analyses focused on data from a random sample of participants in the NutriNet-Santé cohort who had completed the questionnaire assessing their participation motives and who had no missing sociodemographic, lifestyle, anthropometric, or health status data. These characteristics were compared between participants included in our analysis and those who had stopped participating within 6 months after their enrollment (calculated from the date of the last connection on the website), using a chi-square goodness-of-fit test. The possible reasons for participation were grouped into the following 4 categories: (1) to help advance public health research on chronic disease prevention, (2) to help advance nutrition research, (3) in response to the call from the media, from a close friend/associate or from a medical provider, and (4) other motives (ie, to receive regular scientific information about health and nutrition, out of curiosity, to belong to a group, and other). Perceptions/attitudes toward the public funding of the study were categorized into 3 groups: very important, important, and not important.

According to French recommendations [33], male drinkers were categorized as abstainers and irregular consumers (<once a week), moderate (0-30 g alcohol/day), or heavy drinkers (>30 g alcohol/day), and females as nondrinkers (0 g alcohol/day), moderate (0-20 g alcohol/day), or heavy drinkers (>20 g alcohol/day). Normal weight, overweight, and obesity were defined according to the World Health Organization classification for BMI, as BMI <25 kg/m^2^, 25≤ BMI <30 kg/m^2^, and BMI ≥30 kg/m^2^, respectively [34]. Gender, age (≤25, 26-35, 36-45, 46-55, and >55 years), marital status (married or living with a partner, single/divorced/widowed), having at least 1 child at home, education (elementary school, secondary, college graduate, and advanced degree), occupational category (managerial staff, farmers/self-employed, intermediate profession, employees/manual workers, and never-employed/homemaker), type of area of residence (rural, semiurban <20,000 inhabitants, urban 20,000-100,000 inhabitants, urban ≥100,000 inhabitants, Paris metropolitan area), smoking status (never smoker, former smoker, current smoker), alcohol consumption, BMI, self-reported type 2 diabetes, hypertension, and hypercholesterolemia are presented in a frequency/percent format for the whole sample. Multivariate associations between the individual characteristics and the motives for participation (participation motive related to the advantages of the Internet, reasons for participation, and attitudes about public funding) were assessed using multinomial logistic regression. Reference category used in the analysis of the associations between participation motive related to the advantages of the Internet and individual characteristics was yes. For the analysis regarding reasons for participation, the reference category was other motives, and for those concerning attitudes about public funding the reference was not important. In each multivariate model, the explanatory variables were adjusted for each other. Odds ratios (OR) and 95% confidence intervals (CI) are reported. Significance tests were 2-sided with a *P* value set at <.05. A more conservative *P* value of.01 was also used for estimating the robustness of the results. Statistical analyses were performed using SAS software version 9.3 (SAS Institute Inc, Cary, NC, USA).

## Results

A total of 6556 of 15,000 persons completed the motives questionnaire (ie, 43.71% of the randomly invited cohort participants). We excluded 61 individuals with missing data regarding the socioeconomic characteristics, 135 participants with missing data regarding weight or height, and 11 participants with missing data regarding alcohol consumption; therefore, data from 6352 participants was available for analysis. At the time of the administration of the questionnaire about motives, the mean duration of participation in the cohort for the participants included in this analysis was 20 months (SD 4.00) and the median was 23 months (range 1-24). Characteristics of the sample are presented in Table 1.

Compared with nonrespondents (among the 15,000 contacted participants), the percentages of individuals older than 55 years and of managerial staff were higher among participants included in this analysis, whereas the percentage of individuals with at least 1 child at home was lower (data not shown). Compared with participants who stopped participating within 6 months after their inclusion in the cohort (mean duration of participation: 3 weeks after inclusion, SD 1 week), the percentages of individuals older than 45 years, of married persons, managerial staff, persons with high educational level, individuals who reported hypertension, and those who reported hypercholesterolemia were higher among participants included in this analysis, whereas the percentages of individuals with at least 1 child at home, manual workers/employees, infrequent alcohol consumers, smokers, and obese individuals were lower (Table 1).

Among participants, 46.45% (2951/6352) reported that they would not have enrolled had the study not been conducted on the Internet, whereas 28.75% (1826/6352) were not sure (Table 2).

**Table 1:** Characteristics of the sample.^a^

Individual characteristics		Present sample, n (%) n=6352	Drop-out,^b^ n (%) n=9982
**Gender**			
	Female	4821 (75.90)	7584 (75.98)
	Male	1531 (24.10)	2398 (24.02)
**Age (years)**			
	18-25	480 (7.56)	1482 (14.85)
	26-35	1133 (17.84)	2630 (26.35)
	36-45	1211 (19.06)	2276 (22.80)
	46-55	1344 (21.16)	1843 (18.46)
	>55	2184 (34.38)	1750 (17.54
**Marital status**			
	Married or living with a partner	4680 (73.68)	6739 (67.51)
	Single, divorced, widowed	1672 (26.32)	3243 (32.49)
**Have at least 1 child at home**			
	Yes	1976 (31.11)	3946 (39.53)
	No	4376 (68.89)	6036 (60.47)
**Educational level**			
	Advanced/graduate degree	2031 (31.98)	2414 (24.18)
	College graduate	1868 (29.41)	2856 (28.61)
	Secondary	2233 (35.15)	4261 (42.69)
	Elementary school	220 (3.46)	451 (4.52)
**Occupational category**			
	Managerial staff	2215 (34.87)	2437 (24.41)
	Self-employed, farmer	198 (3.12)	424 (4.25)
	Intermediate profession	1673 (26.34)	2101 (21.05)
	Employee, manual worker	1959 (30.84)	4389 (43.97)
	Never-employed/homemaker	307 (4.83)	631 (6.32)
**Area of residence**			
	Rural	1393 (21.98)	2014 (20.18)
	Semiurban, population <20,000	997 (15.71)	1445 (14.48)
	Urban, population between 20,000-100,000	784 (12.39)	1243 (12.45)
	Urban, population ≥100,000	2118 (33.23)	3408 (34.14)
	Urban, Paris	1060 (16.69)	1872 (18.75)
**Alcohol consumption**			
	Abstainers and infrequent consumers (<once a week)	1635 (25.74)	2920 (29.25)
	Moderate consumption (≤20 g/day for women and ≤30 g/day for men)	4192 (65.99)	6308 (63.19)
	Heavy consumption (>20 g/day for women and >30 g/day for men)	525 (8.27)	754 (7.55)
**Smoking status**			
	Never smoker	3195 (50.30)	4374 (43.82)
	Former smoker	2200 (34.63)	2858 (28.63)
	Current smoker	957 (15.07)	2750 (27.55)
**Weight status (BMI)**			
	Normal (<25 kg/m^2^)	4410 (69.43)	6461 (64.73)
	Overweight (≥25 kg/m^2^-30 kg/m^2^>)	1382 (21.76)	2262 (22.66)
	Obese (≥30 kg/m^2^)	560 (8.82)	1259 (12.61)
Self-reported type 2 diabetes (yes)		157 (2.47)	206 (2.06)
Self-reported hypertension (yes)		876 (13.79)	982 (9.84)
Self-reported hypercholesterolemia (yes)		755 (11.89)	678 (6.79)

^a^All *P* values were <.05.

^b^Individuals who stopped participating within 6 months after their inclusion in the cohort.

**Table 2:** Motives for participation in the NutriNet-Santé cohort study (N=6352).

Motives for participation		n (%)
**What was your main reason to participate in the study?**		
	To help advance nutrition research	1413 (22.24)
	To help advance public health research on chronic disease prevention	3898 (61.37)
	In response to the call for volunteers (from media, a friend/associate or a medical provider)	438 (6.89)
	Other^a^	603 (9.50)
**Would you have participated in the study if it were not Internet-based?**		
	Yes	1575 (24.80)
	No	2951 (46.45)
	Don’t know	1826 (28.75)
**Is the fact that the study is exclusively funded by public sources important for your participation?**		
	Very important	2185 (34.40)
	Important	2072 (32.62)
	Not important	2095 (32.98)

^a^Other category includes participation to receive regular scientific information about health and nutrition, out of curiosity, to belong to a group, and other.

Compared to women, men were more inclined to be motivated by the Internet aspect (Table 3). Individuals aged between 26 and 35 years, those without children at home and obese persons also displayed increased motivation associated with the Internet aspect. Individuals older than 55 years, employees/manual workers, and those in intermediate professions were less likely to be motivated by the Internet aspect compared to younger adults and managerial staff (Table 3). Results regarding occupational categories and having at least 1 child at home did not remain significant when the more conservative *P* value of.01 was used.

**Table 3:** Sociodemographic, lifestyle, and health characteristics associated with motives for participation in the study had it not been Internet-based (multivariate analysis, N=6352)

Individual characteristics		No, I would not have enrolled^a^		I don’t know^a^	
		OR	95% CI	OR	95% CI
**Gender**					
	Female	1.00		1.00	
	Male	1.22	1.04-1.43	0.96	0.80-1.14
**Age (years)**					
	18-25	1.21	0.86-1.70	1.19	0.82-1.73
	26-35	1.51	1.20-1.91	1.39	1.08-1.79
	36-45	1.00		1.00	
	46-55	0.90	0.74-1.11	0.86	0.68-1.07
	>55	0.61	0.49- 0.76	0.76	0.59-0.96
**Marital status**					
	Married or living with a partner	1.00		1.00	
	Single, divorced, widowed	0.97	0.83-1.13	1.00	0.85-1.18
**Have at least 1 child at home**					
	Yes	1.00		1.00	
	No	1.23	1.04-1.46	1.22	1.02-1.47
**Educational level**					
	Advanced/graduate degree	1.00		1.00	
	College graduate	1.10	0.92-1.31	0.98	0.81-1.19
	Secondary	1.10	0.91-1.33	1.00	0.82-1.24
	Elementary school	1.18	0.80-1.74	1.38	0.92-2.07
**Occupational category**					
	Managerial staff	1.00		1.00	
	Self-employed, farmer	1.09	0.74-1.60	1.14	0.75-1.75
	Intermediate profession	0.80	0.67-0.96	1.04	0.85-1.26
	Employee, manual worker	0.77	0.63-0.93	0.89	0.72-1.10
	Never-employed/homemaker	1.03	0.69-1.55	1.21	0.78-1.87
**Area of residence**					
	Rural	1.00		1.00	
	Semiurban population <20,000	1.01	0.83-1.23	1.07	0.85-1.34
	Urban, population between 20,000-100,000	0.81	0.65-1.01	0.85	0.67-1.08
	Urban, population ≥100,000	0.91	0.77-1.08	0.98	0.81-1.18
	Urban, Paris	1.00	0.81-1.23	1.03	0.82-1.29
**Alcohol consumption**					
	Abstainers and infrequent consumers (<once a week)	1.00			
	Moderate consumption (≤ 20 g/day for women and ≤30 g/day for men)	1.18	1.02-1.37	1.20	1.02-1.40
	Heavy consumption (>20 g/day for women and >30 g/day for men)	1.19	0.92-1.53	0.98	0.74-1.31
**Smoking status**					
	Never smoker	1.00		1.00	
	Former smoker	0.97	0.84-1.12	1.01	0.86-1.18
	Current smoker	0.94	0.78-1.13	1.01	0.82-1.24
**Weight status (BMI)**					
	Normal (<25 kg/m^2^)	1.00		1.00	
	Overweight (≥25 kg/m^2^-30 kg/m^2^>)	1.07	0.91-1.25	1.09	0.92-1.30
	Obese (≥30 kg/m^2^)	1.32	1.04-1.65	1.44	1.11-1.86
Self-reported type 2 diabetes (yes)		0.90	0.61-1.34	0.70	0.44-1.10
Self-reported hypertension (yes)		1,06	0.87-1.28	0.92	0.74-1.14
Self-reported hypercholesterolemia (yes)		1.05	0.86-1.28	1.04	0.83-1.30

^a^The question was “Would you have participated in the study if it were not Internet-based?” Reference category for the outcome variable was “Yes, I would still participate even if the study was not Internet-based.”

Regarding reasons for participation, 61.37% (3898/6352) reported participating to help advance public health research on chronic disease prevention; 22.24% (1413/6352) to help advance nutrition-focused research; 6.89% (438/6352) in response to a call from the media, a close friend/relative, or a medical professional; and 9.50% (603/6352) for other reasons (Table 2). Unlike younger participants, individuals older than 45 years were more likely to participate to help advance public health research on chronic disease prevention and to help advance nutrition research (Table 4). Overweight or obese persons were less inclined to participate for these reasons compared with individuals with normal weight. Single, divorced, or widowed individuals were less likely to participate for reasons of helping nutrition research or in response to the call from the media, a close friend/relative, or a medical professional than were individuals living with a partner. Finally, never-employed/homemakers were less likely to participate to help advance public health research on chronic disease prevention than were managerial staff.

Exclusive public funding for the study was important for two-thirds of the participants. Among them, half (2185/6352, 34.40%) considered it as very important (Table 2). Compared to women and to individuals aged between 36 and 45 years, men and persons older than 45 years were more likely to consider the exclusive public funding as very important or important, whereas younger participants were less likely to find it very important or important (Table 5). Compared to persons with the highest educational level, managerial staff and those with at least 1 child at home, individuals with less formal education, self-employed/farmers, employees/manual workers, or those without children at home were less likely to consider the exclusive public funding as very important or important. Results regarding having at least 1 child at home did not remain significant when the more conservative *P* value of .01 was used.

**Table 4:** Sociodemographic, lifestyle, and health characteristics associated with reasons for participation in the study (multivariate analysis, N=6352).

Individual characteristics		To help advance public research on chronic disease prevention^a^		To help advance nutrition research^a^		In response to the call (from the media, a friend/associate or a medical provider)^a^	
		OR	95% CI	OR	95% CI	OR	95% CI
**Gender**							
	Female	1.00		1.00		1.00	
	Male	1.02	0.82-1.27	0.86	0.67-1.09	0.96	0.70-1.32
**Age (years)**							
	18-25	1.00		1.00		1.00	
	26-35	1.08	0.72-1.61	1.43	0.91-2.25	1.05	0.59-1.89
	36-45	1.31	0.85-2.01	1.55	0.96-2.51	1.00	0.53-1.87
	46-55	1.63	1.07-2.48	1.74	1.09-2.79	1.48	0.81-2.69
	> 55	1.62	1.07-2.46	1.43	0.90-2.29	1.33	0.73-2.41
**Marital status**							
	Married or living with a partner	1.00		1.00		1.00	
	Single, divorced, widowed	0.82	0.67-1.01	0.79	0.63-0.99	0.69	0.51-0.94
**Have at least 1 child at home**							
	Yes	1.00		1.00		1.00	
	No	1.02	0.82-1.29	0.9	0.70-1.15	1.08	0.78-1.49
**Educational level**							
	Advanced/graduate degree	1.00		1.00		1.00	
	College graduate	1.01	0.79-1.28	1.17	0.90-1.52	1.17	0.82-1.66
	Secondary	1.13	0.86-1.47	0.97	0.72-1.31	1.21	0.82-1.78
	Elementary school	1.39	0.80-2.40	0.66	0.34-1.29	1.26	0.59-2.72
**Occupational category**							
	Managerial staff	1.00		1.00		1.00	
	Self-employed, farmer	1.51	0.79-2.89	1.51	0.75-3.04	2.23	0.99-5.00
	Intermediate profession	0.97	0.75-1.25	0.89	0.68-1.18	1.02	0.71-1.48
	Employee, manual worker	0.85	0.65-1.10	0.75	0.56-1.00	1.14	0.78-1.67
	Never-employed/homemaker	0.56	0.35-0.91	0.77	0.46-1.31	0.98	0.49-1.96
**Area of residence**							
	Rural	1.00		1.00		1.00	
	Semiurban, population <20,000	1.28	0.95-1.72	1.15	0.83-1.60	1.03	0.68-1.56
	Urban, population between 20,000-100,000	0.99	0.73-1.34	1.03	0.73-1.44	1.01	0.66-1.56
	Urban, population ≥100,000	0.96	0.76-1.21	0.95	0.73-1.23	0.91	0.65-1.28
	Urban, Paris	1.28	0.95-1.72	1.07	0.77-1.48	1.12	0.74-1.70
**Alcohol consumption**							
	Abstainers and infrequent consumers (<once a week)	1.00		1.00		1.00	
	Moderate consumption (≤20 g/day for women and ≤30 g/day for men)	0.84	0.68-1.03	0.84	0.66-1.06	0.80	0.60-1.08
	Heavy consumption (>20 g/day for women and >30 g/day for men)	0.85	0.59-1.22	0.84	0.56-1.26	0.80	0.48-1.34
**Smoking status**							
	Never smoker	1.00		1.00		1.00	
	Former smoker	0.88	0.72-1.08	0.83	0.66-1.04	0.83	0.62-1.11
	Current smoker	0.91	0.71-1.18	0.96	0.72-1.27	1.02	0.71-1.47
**Weight status (BMI)**							
	Normal (<25 kg/m^2^)	1.00		1.00		1.00	
	Overweight (≥25 kg/m^2^-30 kg/m^2^>)	0.72	0.58-0.89	0.72	0.57-0.92	0.94	0.69-1.28
	Obese (≥30 kg/m^2^)	0.62	0.46-0.84	0.71	0.50-1.00	0.80	0.52-1.24
Self-reported type 2 diabetes (yes)		1.77	0.87-3.60	1.59	0.73-3.49	2.05	0.84-5.01
Self-reported hypertension (yes)		0.83	0.63-1.09	0.81	0.59-1.11	0.83	0.56-1.23
Self-reported hypercholesterolemia (yes)		1.30	0.95-1.77	1.23	0.87-1.74	1.08	0.70-1.68

^a^The question was “What was your main reason to participate in the study?” Reference category for the outcome variable was “other reasons” which includes participation to receive regular scientific information about health and nutrition, out of curiosity, to belong to a group, and other.

**Table 5:** Sociodemographic, lifestyle, and health characteristics associated with importance for exclusive public funding (multivariate analysis, N=6352).

Individual characteristics		Very important^a^		Important^a^	
		OR	95% CI	OR	95% CI
**Gender**					
	Female	1.00		1.00	
	Male	1.37	1.17-1.61	1.21	1.03-1.42
**Age (years)**					
	18-25	0.40	0.28-0.58	0.57	0.41-0.78
	26-35	0.63	0.51-0.79	0.79	0.64-0.97
	36-45	1.00		1.00	
	46-55	1.49	1.21-1.83	1.14	0.93-1.40
	> 55	1.97	1.57-2.47	1.37	1.09-1.71
**Marital status**					
	Married or living with a partner	1.00		1.00	
	Single, divorced, widowed	0.96	0.82-1.12	1.09	0.94-1.26
**Have at least 1 child at home**					
	Yes	1.00		1.00	
	No	0.81	0.68-0.95	0.94	0.80-1.11
**Educational level**					
	Advanced/graduate degree	1.00		1.00	
	College graduate	0.80	0.67-0.96	0.92	0.77-1.10
	Secondary	0.41	0.34-0.50	0.61	0.50-0.74
	Elementary school	0.38	0.26-0.57	0.63	0.44-0.92
**Occupational category**					
	Managerial staff	1.00		1.00	
	Self-employed, farmer	0.63	0.43-0.93	0.87	0.61-1.26
	Intermediate profession	0.87	0.73-1.04	0.90	0.75-1.08
	Employee, manual worker	0.54	0.44-0.65	0.71	0.59-0.86
	Never-employed/homemaker	0.76	0.50-1.15	1.16	0.81-1.66
**Area of residence**					
	Rural	1.00		1.00	
	Semiurban, population <20,000	1.02	0.83-1.26	1.06	0.86-1.30
	Urban, population between 20,000-100,000	0.95	0.76-1.18	0.93	0.75-1.16
	Urban, population ≥100,000	1.08	0.91-1.29	1.04	0.88-1.24
	Urban, Paris	0.97	0.78-1.19	1.01	0.82-1.24
**Alcohol consumption**					
	Abstainers and infrequent consumers <once a week)	1.00		1.00	
	Moderate consumption ≤20 g/day for women and ≤30 g/day for men)	0.99	0.86-1.16	0.97	0.84-1.13
	Heavy consumption >20 g/day for women and >30 g/day for men)	0.78	0.60-1.02	1.00	0.78-1.29
**Smoking status**					
	Never smoker	1.00		1.00	
	Former smoker	1.08	0.93-1.25	1.12	0.97-1.30
	Current smoker	1.12	0.93-1.35	1.03	0.86-1.24
**Weight status (BMI)**					
	Normal <25 kg/m^2^)	1.00		1.00	
	Overweight ≥25 kg/m^2^-30 kg/m^2^>)	0.89	0.76-1.05	0.92	0.78-1.08
	Obese ≥ 30 kg/m^2^)	0.80	0.63-1.01	0.78	0.62-0.98
Self-reported type 2 diabetes (yes)		1.06	0.69-1.61	0.92	0.60-1.42
Self-reported hypertension (yes)		1.16	0.94-1.42	1.13	0.92-1.39
Self-reported hypercholesterolemia (yes)		1.13	0.91-1.40	1.18	0.95-1.46

^a^The question was “Is the fact that the study is exclusively funded by public sources important for your participation?” Reference category for the outcome variable was “not important.”

## Discussion

### Principal Results

Profiles of participants in a Web-based epidemiological cohort have rarely been studied [8,10-16] and motives to participate in such cohorts have not yet been explored. The present study highlighted specific sociodemographic and health status (ie, weight status) characteristics of participants in a large Web cohort according to the perceived importance of the Internet, the reasons for participation, and the importance of public funding for research. Our results could guide the development of novel strategies for the retention of diverse population samples in Web-based cohorts, particularly during key periods of data collection.

Our findings revealed that almost half of the participants reported that Internet use was a decisive reason for participation. In fact, this element exerted a stronger influence among men, persons younger than 35 years, individuals of higher socioeconomic status, those without children at home, moderate alcohol consumers, and obese persons. Our results are concordant with previous studies that compared sociodemographic and lifestyle characteristics of participants who used Web-based questionnaires with those of participants who used paper-and-pencil instruments [8,14]. There is clear evidence that men, young people, and single persons are less likely to participate in epidemiological studies than are women, older, or married individuals [9,35-37]. Next, several studies have shown that individuals who presented risk behaviors, such as smoking, heavy alcohol use, or obesity, were often underrepresented among research participants [9,38,39]. Thus, our study suggests that the Internet allows for reaching a large population, but also provides access to hard-to-reach individuals given their social or behavioral status and for whom the Internet seems to be a more attractive and more convenient mean for participation compared to traditional means. Individuals belonging to low socioeconomic strata are often poorly represented when relying on traditional methods [40]. Our findings showed that the Internet appeared to be a less important motive for participation among individuals in low occupational categories, compared to managerial staff. Therefore, further exploration of measures that can be adopted in epidemiological Web-based studies to specifically increase opportunities for participation among low socioeconomic groups is necessary.

In addition, our study indicated that participation in an exclusively Web-based nutrition cohort was driven mainly by a desire to help advance research on chronic disease prevention or nutrition, especially among older participants, those with normal weight, and those who lived with a partner. Our results are also consistent with existing knowledge regarding motives for participation in volunteer-based cohorts on health and nutrition, which do not use the Internet [24,26-28]. These reasons for participation may reflect altruistic tendencies, but also a vested personal interest [27]. As explained by Williams et al [28], individuals may be more willing to participate if they believe that the potential benefits of their participation are large (eg, life versus death), highly likely to materialize, quickly attained, or likely to benefit themselves or someone important to them. Our results suggest that the desire to contribute to chronic disease risk prevention should be used as a key lever for participants’ retention in Web-based cohorts, particularly during decisive periods of exposure data collection.

Two-thirds of our sample found the use of Internet for completion of the questionnaires to be a benefit, given its flexibility, whereas less than 1% found it to be complex. Also, one-quarter of participants felt more comfortable filling in the questionnaires on the website rather than face to face with an investigator. On the other hand, only 22% visited sections of the website of the study regarding news and progress of the study. Thus, in Web-based studies, the reduced participant burden (eg, quick, easy and convenient access, increased distance between participant and investigator allowing participants not to feel judged) [41] should help minimize attrition.

Participation in the study for altruistic reasons may be reinforced by the public nature of the research. Indeed, two-thirds of the participants considered the exclusive public funding as important or very important, with the link being particularly pronounced in men, older adults, and individuals of higher socioeconomic status. This finding is not surprising in a European context in which the majority of cohort studies are funded by public organizations. Indeed, 94% of French participants in an opinion survey conducted in the general French population reported that a large part of biomedical research needs to be funded by public funding, and 80% of responders feared that the increased participation of private funding in public research could undermine the independence of research and is damaging to certain research areas deemed less profitable [42]. In turn, the use of Internet for epidemiological studies could be viewed with suspicion by some participants because of fear that personal information might be diffused or sold to third parties or that their responses might not be anonymous [43]. In addition to reassuring participants that their personal information is kept private and safe, it seems important to point out the fact that the study is exclusively funded by public sources to investigate important public health issues.

### Limitations

Our study has several limitations. First, responders were older and belonged to higher socioeconomic strata than nonrespondents, which might have led to an underestimation of the observed associations. Moreover, our result suggests that the influence of weight status on participation is open to question because participants in a long-term cohort are likely to be particularly health conscious and interested in nutritional issues. In addition, results may reflect the motives of participants accustomed to the study rather than their motives for enrollment in the Web-based cohort because the questions about motives were asked approximately 2 years after baseline. However, key information on exposure and potential confounding factors was collected during the first 2 years of participation in the cohort. Thus, a focus on the motives of those participants who actively participated 2 years after their inclusion is useful in terms of retention strategies during decisive periods of data collection in Web-based cohorts. In addition, the percentage distributions of the given reasons for participation could be biased due the use of a predefined list of response options. However, the participants had the opportunity to choose the “other” response option and to specify the exact reason for participation. Another limitation was the lack of information on reasons for declining participation because the call for participation was not delivered to a predefined list of individuals. Finally, anthropometric status was assessed by self-reporting and may have led to misclassification. However, in a separate validation study that used data from a subsample of 2513 participants, we compared self-reported and measured height and weight (and the resulting BMI) [44]. In particular, these participants had completed the anthropometric questionnaire 3 days before the clinical examination. The intraclass correlation coefficient ranged from .94 for height to .99 for weight. BMI classification was correct in 93% of the cases; the weighted kappa statistic was .89. Of 2513 participants, 23.5% were classified as overweight (BMI ≥25) with Web-based self-report versus 25.7% based on measurements by study staff, leading to a sensitivity of 88% and a specificity of 99%. For obesity, 9.1% versus 10.7% were classified as obese (BMI ≥30), respectively, leading to sensitivity of 83% and specificity of 100%.

### Conclusions

Our study highlighted that the reliance on the Internet, the willingness to help advance public health research, and the exclusive public funding of the study were key motives for participation in this exclusively Web-based cohort. In addition, these motives for participation differed by sociodemographic profile and BMI, but not by lifestyle or health status. These findings can help improve retention of diverse population samples, particularly during important data collection periods.

## Abbreviations

BMI: body mass index
